# Creative leaps in theory: the might of abduction

**DOI:** 10.1007/s10459-021-10057-8

**Published:** 2021-06-17

**Authors:** Mario Veen

**Affiliations:** Department of General Practice, Erasmus Medical Center Rotterdam, Postbus 2040, 3000 Rotterdam, CA The Netherlands

**Keywords:** Abduction, Theory, Research

## Abstract

This paper argues that abductive reasoning has a central place in theorizing Health Professions Education. At the root of abduction lies a fundamental debate: How do we connect practice, which is always singular and unique, with theory, which describes the world in terms of rules, generalizations, and universals? While abduction was initially seen as the ‘poor cousin’ of deduction and induction, ultimately it has something important to tell us about the role of imagination and humility in theorizing Health Professions Education. It is that which makes theory possible, because it allows us to ask what *might* be the case and calls attention to the role of creative leaps in theory. Becoming aware of the abductive reasoning we already perform in our research allows us to take the role of imagination—something rarely associated with theory—seriously.

## Abduction’s might

We need more abduction in HPE research. Not the criminal kind, but rather, one that allows us to enter into a dialogue between theory and practice as a way to produce new ideas for our field.

I open the fridge to take out the carton of milk I bought yesterday, but it is not there. Since only my wife and I are at home, I logically conclude she might have drunk it. This is an instance of abductive reasoning. Someone might have broken into my house and stolen the milk. I might have woken up thirsty during the night and forgotten about drinking it myself. The operative word here is *might.* There are other explanations that *might* be possible, but they are even less likely.

Abduction is a form of logical inference that was introduced to the philosophical canon by the philosopher Peirce (1903, 2014; see also Douven 2017). Abduction describes something we do all the time: inferring the best possible explanation for what we observe. For example, when I am in a shop and I cannot find something, I look for someone who is dressed as if they might work there. Educators also use abduction. A teacher observes that a trainee, for instance, is staring into the distance with a pensive demeanor and they conclude that the trainee is reflecting. The trainee who is indeed reflecting, is considering various possible explanations for an error they just made. These are both instances of abduction.

In abduction, we begin with an observation and extrapolate to a conclusion—e.g. that my wife has drunk the milk, that someone is an employee of a shop, that a student is reflecting. Abduction is the mechanism via which we generate hypotheses about what we observe. It formulates a possible cause for an observable result, and as such introduces a creative element into our reasoning. This newness is what distinguishes abduction from other forms of reasoning and what, according to Peirce, makes innovation in theory possible.

We routinely use abduction in health professions education (HPE) and HPE research. For instance, I may observe a trainee arriving late for class and abduct that the trainee is unprofessional. While that may indeed be the case, however, it may also be the case that the trainee was late because they spent extra time with a patient making sure that the care plan fit the patient’s needs and expectations. As this example illustrates, although we use abduction routinely, abductive reasoning is often carried out intuitively, based on individual perceptiveness and available information. As such, the work of abductive reasoning is rarely made explicit and it is not subject to scrutiny like other forms of reasoning.

Although we may sometimes view theory formation in HPE research as a purely rational process, abduction is actually based on perceptiveness, imagination, and creativity. We must first *notice* that something is the case, which means that we should be open to something (a line of a transcript we are coding, a badge on a shirt, an empty glass by my beside) that stands out from its surroundings. We must then be able to *imagine* that it is likely to be a case of something, even if it is something we have not yet considered. Finally, we create a coherent whole from these different elements, drawing on other forms of reasoning, such as induction, deduction, and metaphoric inference.

Although it is difficult to give a precise definition of abduction, I will begin to delineate it further by discussing what it adds to two better known types of logical inference: deduction and induction. I will then discuss how abduction is a fundamental trait of all theory because it goes to the heart of an essential job of theory: to see something *as* something else. By discussing abduction in the context of diagnostic reasoning, we will be better able to understand the role played by perceptiveness (as affect or intuition) and imagination. I will then show how abduction is indeed relevant for all levels of theory in HPE research, demonstrating how it is used in HPE research during data analysis and at those moments when we connect practice to theory by operationalizing or translating complex concepts. Finally, I will conclude that abduction shows us the necessity of both a sense of humility, and the potential of creative leaps in HPE theory.

## The logic of abduction

When originally developed as a formal approach to reasoning, abduction was described as an addition to two categories of logical inference: deduction and induction. In the context of theory formation, we work with three elements of logical inference. The first is an observation or measurement, which we call the *result*. A result is a consequence of a cause. The second is a general principle or universality, which we call the *rule*. A rule is an abstraction, for instance, a cause that we cannot perceive. It is not a percept (something we can directly observe), but an inference. The third describes the relationship between the result and the rule: The result is an instance or *case* of a rule. This last element, treating percepts as cases of general principles, lies at the heart of theory formation.

In the example of the store, we abduce that the person is dressed in a certain way as a consequence of being an employee. However, we could equally be wrong, and they may be dressed that way for other reasons. In this example, induction would express a situation in which we enter a store, observe that four out of the ten people present are dressed exactly the same, and generalize that they must be employees. Employing deduction, we start with the rule that employees wear name badges or operate the till and look for evidence that fits these deductions.

Deduction, induction, and abduction differ in how they relate these elements and in which order. I will use the classic example of the beans to illustrate this. The following example is adapted from Svennevig (2001).

Deductive reasoning is the development of a conclusion based on generally accepted premises, statements, or facts. It is the construction of a conclusion from an observation because we treat the observation as a case of rule.

- *Rule (or hypothesis)*: All the beans in this bag are black.
- *Case*: The beans in my hand are from this bag.
- *Result*: The beans in my hand are black.

Inductive reasoning is the development of conclusions from a series of individual observations. It is the creation of a general rule from specific instances.

- *Case (or sample)*: I take a handful of beans from this bag and do this a few times.
- *Result*: They are all black.
- *Rule*: All the beans in this bag are likely to be black.

Abduction is an aspect of inferential reasoning that escapes deduction and induction because both are hypothetical in nature. In deductive reasoning, how do we arrive at the initial premises of deduction? In deductive reasoning, how do we make the leap from recording multiple observations to inferring that these observations are part of a pattern? If we revisit the bag of beans example, we can construct an example of abduction:

- *Result (or observation)*: There are some black beans on the table.
- *Rule*: Next to the beans sits a bag labeled ‘black beans’.
- *Case*: The beans on the table might be from that bag.

There are two things to notice about abduction in relation to induction and deduction. First, abduction generates a probable conclusion. It is possible, albeit unlikely, that the beans in our example came from somewhere else, perhaps another bag. Abduction, therefore, never provides certainty because there are always other possible explanations. Abduction has an inherent humility because it acknowledges that, while the conclusion is a sufficient explanation of the observation, it is not necessarily the *only* possible explanation. Indeed, there may be other rules that would explain the case just as well, or even better.

Second, abduction leads to a different kind of conclusion than either deduction or induction. In deduction, we infer or predict a *result*. Black beans are labeled ‘black beans’ in the store, so we might deduce that if you grab a handful of beans from the bag, the result will be that you have a handful of black beans. There is no need to even look before you throw them in the pan. In induction, you infer a *rule*. Taking a few samples from an unlabeled bag leads you to generate the rule that ‘all beans in this bag are black’. In abduction, the conclusion about the observation is neither a result nor a rule, but a *case*, i.e., you conclude that the instance is *a case of* a more general rule.

To summarize, deductive and inductive conclusions are presented as the only (or most) correct theory. In induction, the conclusion is presented as a rule that is necessarily ‘given’ by the observations made. In abduction, the conclusion we arrive at is *a* theory, not *the* theory. It is *a* case, but not the *only* case. Abduction provides possible accounts for why something *might* be the case.

## Abduction as a fundamental trait of all theory

At this point, we might think that we need to decide what kind of approach we want to take to theory in our research: deductive, inductive, or abductive. However, abduction does not so much indicate a *type* of theory formation, as an *aspect* of all theory formation. In addition, logical inference is not only at play at the macro-level of theory formation, where we formulate a hypothesis and test it experimentally (deduction) or use an ‘inductive’ approach such as grounded theory. It is present at the meso-level as well, for instance, when we relate different findings of our research to journal articles, or when we decide the point at which we have reached ‘data saturation’. It is also present at the micro-level, where we decide to assign *this* code to *this* sentence in our interview transcript or perform *this* statistical operation in SPSS. Peirce even went so far as to state that whenever we go beyond merely observing data and treat a concrete observation as an instance of an abstract category, we are making an abduction: “Neither deduction nor induction can ever add the smallest item to the data of perception; [not] the smallest advance can be made in knowledge beyond the stage of vacant staring without making an abduction at every step” (Peirce 1901, p.900).

Abduction, therefore, goes to the heart of theory and precedes both induction and abduction. A theory is a set of interrelated and well-substantiated ideas intended to explain something. Theories systematically connect concepts (such as gravity), relationships or processes (such as causation), and percepts (phenomena that we perceive ‘to be the case’, such as apples falling). When we think of theory in relation to ways of reasoning, theory can be thought of as a frame through which we come to classify or understand a singular instance as a case of a general rule. Why else would we use theory in HPE, if not to make informed choices about the way we go about teaching and learning, analyze whether these choices worked, and use this feedback from practice to constantly improve our theory? The case of the trainee arriving late to class can be understood as a single case that we classify via the frame of theories of unprofessional behavior. As this illustration highlights, “theories are intimately connected to practical experiences” (Varpio and Ellaway 2021, p. 341). This does not mean, however, that all theories we use in practice are easily identified. Kant (1996) showed that there are theories without which we are unable to experience anything at all, such as time, space, and causation. Furthermore, as Popper (2002) explained, a good theory includes interactions—sometimes even violent clashes—with experience that result in the falsification of the theory.

At their most basic, theories are inventions. No matter how formal, intricate, and precise, theories are narratives that humans invent to make sense of life, for instance, by predicting that X always happens when I do Y. The building blocks of theory are linguistic, if we includ the universal language of mathematics and the probabilistic language of statistics. Theories propose ways of seeing something in the world, perhaps an entire field, *as* something else, in order to act (or refrain from acting) on it in a certain way. The *as* allows us to make an observation and to interpret it beyond saying: ‘It is what it is’ (Peirce’s “vacant staring” (1901)). Theory allows us to see something a trainee does (e.g., arrive late for education) *as* unprofessional. When we analyze an interview with a medical trainee as part of a HPE study, we are often not interested in the trainee *as* a mother or a father, *as* a person with a unique history and personality, or *as* someone with whom we want to become friends. Rather, depending on our research aim, we treat the data as an instance of a larger, more abstract phenomenon (e.g., a social value, a professional group, a rule, or a condition).

Deduction and induction construct conclusions that are limited by their initial premises and observations respectively. Deduction only permits us to connect observations to already known results or theories; induction only permits us to infer rules or theories by combining observations. A foundational concern arises from these limitations: How do we come up with the initial hypothesis? In deduction, we do not just randomly choose hypotheses that we test. There is something ‘in me’ that tells me that *this* theory, proposed by *this* journal article, is promising, or is to be preferred over other theories. Moreover, when we make observations, how do we generate an explanation for those observations? The observations themselves do not generate anything. Inductive reasoning is not simply a list or series of observations: ‘I observed X, then I observed Y, then I observed Z’. Instead, the observations lead to a rule, however, how do we know which rule to construct?

Arriving at hypotheses, combining affects with the imagination of possible explanations, and formulating rules on the basis of observations, are the work of abduction. Therefore, I would argue that abduction lies at the root of all the explanatory force that results from theory. Abduction is at the root of all conclusions that go beyond mere descriptions of what can be observed. Although abduction starts with an observation, instead of inducing a theory about the observations or deducing their meaning from a previously established theory, the abductive question asks: ‘How *might* I interpret this observation?‘

## Abduction in diagnostic reasoning and the role of affect

To explore the role of abduction in HPE research, we can consider the ways in which healthcare trainees are taught to form theories from observations of their patients. Literature on medical diagnostic reasoning often draws analogies between diagnostic reasoning and fictional detectives such as Sherlock Holmes. However, “[a]lthough he describes his method as ‘deductive’, Holmes did not generally apply either deduction (from the general to the particular) or induction (from particular to general) but rather abduction” (Rapezzi et al. 2005, p.331). Similarly, physicians are primarily concerned with constructing hypotheses that account for their patients’ symptoms (Magnani 1992; Rapezzi et al. 2005; Vertue and Haig 2008). In the process of differential diagnosis, for instance, physicians rely on abductive reasoning to distinguish between multiple possible reasons for specific symptoms. ‘What if we treat this stomachache *as* a symptom of condition A, B or C?’ This may be followed by a theory-empiricism cycle (Varpio and Ellaway 2021): ‘If the patient has condition A, then I would also expect symptom X to be present. I do not see symptom X, therefore we can eliminate condition A.’ Next, we might select another likely diagnosis and attempt to verify it. This could take the form of a question: Did you eat something unusual today? The patient could answer that they ate a pile of black things that were beside a bag of black beans, so they abductively inferred that they were black beans, but they may not have been black beans. This could account for their stomach pain. As this example illustrates, the daily work of physicians often involves imagining which ‘bag’ (medical condition or rule) the ‘beans’ they encounter (observations about their patients as symptoms of conditions) might be from.

This example demonstrates the role of perceptiveness in abduction, both in terms of positive, observable phenomena, and in terms of that which is *not* observed. Experienced physicians will notice the obvious symptoms presented by the patient. They will also observe that which is *absent* from the patient’s narrative, analogous to Homes’ noticing that the dog did not bark in the nighttime. Yet how do we notice something? This is a matter of affect. Deduction only starts when we have already have rules or premises to work from. Induction only starts when we have observed something. Logic can construct conclusions from premises, but it cannot select which premises are (or are not) relevant in each case. In abductive reasoning, theory offers awareness of what is *not* happening, and of what *affects* us. However, there are always many things *not* happening and many things *affecting* us. To identify one absence as *relevant* is clearly not something ‘the world tells us’, or something we ‘logically infer’, but instead requires human imagination.

In literature on diagnostic reasoning, the role of intuition and ‘gut feelings’ (Stolper et al. 2009) has been increasingly acknowledged. Instead of only analyzing medical situations inductively or deductively, physicians are also *affected* by those situations. A hunch may lead to the formulation of a hypotheses (abduction), which then needs to be empirically tested (deduction) and verified (induction). A surprising observation may lead the physician to slow down and wonder what else might be the case that they have not noticed (Moulton et al. 2007). Abduction challenges the view that analytic reasoning proceeds by strict induction and deduction, and that the moment any kind of creative leap comes into play, we have left the world of logic. Affect—in the form of imagination, creativity, surprise, intuition, and perceptiveness—is a part of logical reasoning, and therefore of theory formation.

## The practice of abduction in HPE research

At the root of abduction lies a fundamental debate: How do we connect practice, which is always singular and unique, with theory, which describes the world in terms of rules, generalizations, and universals? A general consensus in HPE research is that we should start with practice: the reality of the education that trainees, educators, and staff encounter every day. The second thing we tend to agree upon is that practice is messy. There are always contextual factors that we cannot take into account and models of practice are always idealized. So, how can we go from an observation of a single case (or multiple observations of multiple cases) to a theory? This requires that we neither apply theories, nor jump from one case study to generalizations, but rather that we move “back and forth between one special view and another” (Bal 2022).

Let us consider the use of recordings of interactions, such as interviews with participants, focus groups, or recordings of education sessions, as a basis for theorizing. Studies that code according to pre-existing frameworks are often considered deductive, while approaches that are data-driven are presented as inductive. However, as Svennevig (2001) claims, if we look, not at what scholars say about their method, but at what they actually do when they are analyzing their data, abduction provides a more accurate description of the research research process. For instance, if “a researcher did not have any expectations of what the data *ought* to look like, there would be no puzzling facts, nothing to explain” (Svennevig 2001, p.7, emphasis in original).

What is it that makes us notice something in our analyses? What makes it stand out from its surroundings (MacLure 2010)? It could be that we notice something because it is surprising: either a surprising single instance, or a surprising pattern—for example that participants keep bringing up topic X with concept Y. However, it can only be surprising if we have an expectation of what is ordinarily the case. Logic can help us work out the implications of a given theory, or construct theories as implications of practice, but it cannot capture what is surprising.

For instance, in my own research on collaborative group reflection I noticed that a tutor did not respond to a trainee’s question (Veen and de la Croix 2016; Veen and Croix 2017). In Conversation Analysis there is a rigid systematic description of what people ordinarily do in conversation, and a cyclical analytic procedure where the researcher alternates between analyzing details of the interaction, viewing the dataset as a whole, and integrating insights from relevant literature on Conversation Analysis along the way. From this literature (Sidnell and Stivers 2012), I know that in ordinary conversation, the ‘rule’ is that when someone asks a question the respondent tries to answer it. If I notice in studying a video of collaborative reflection meetings that, instead of answering a trainee’s question, the tutor instead shifts their gaze to the other members of the group, this stands out as an unexpected act.

If this were familiar then we might deduce why this had happened, or we might infer what the tutor intends from other observations. Instead, I require to generate a likely explanation for the tutor’s gaze shift. Perhaps the tutor did not hear the question. Perhaps the tutor is in a bad mood and does not feel like addressing the question. That is to say, the observation may be a case of collaborative reflection, or of miscommunication, or unique to this particular tutor or this particular group. The question ‘what is this behavior a case of?’ makes this abduction. Over time, it may be possible to rule out some of the possible explanations in favor of a preferred hypothesis. If the tutor does display recognition of the question at a later stage, for instance by acknowledging that another trainee has answered it, we can rule out that they did not hear the question. By analyzing other education sessions, we can see if this way of treating a question by a tutor is indeed unique to that tutor or group. As this example illustrates, abduction happens when I see the tutor’s gaze *as* didactic behavior.

In the process of analyzing other fragments in the dataset, we notice behavior that is not exactly the same, but that we connect, nonetheless. A tutor may interrupt a trainee in the middle of their sentence. We may treat the first and second observations as cases of a hypothesis about collaborative group reflection: Tutors display interactional behaviors that in ordinary conversation would be ‘dispreferred’. Here, I introduce the term ‘dispreferred’ in line with findings from Conversation Analysis (and ethnomethodological research) regarding an orientation toward saving face, to normative orientations (Bilmes 1988). A next step could be to then abduce these observations to the overarching educational goal of the setting in which the interaction takes place: This is a *tutor* in a *reflection group*, whose prescribed institutional role is to facilitate reflection. Could such observations, where tutors act in ‘dispreferred’ ways, have something to do with facilitating that reflection? For example, that not answering a question is a way of prompting the questioner or someone else in the group to address it?

## Creative leaps grounded in practice

As we have seen in the examples of abduction in diagnostic reasoning and HPE research, the question of ‘what is this a case of?’ or ‘what can we see this *as*?’ is central to abduction as well as to theory formation. This requires imagination: ‘what if we treat X as a case of Y?’ But abduction is not mere speculation. Abduction “makes creative leaps [but] its origin in observable fact remains primary.” (Bal 2022) This combination of being rigorously grounded in empirical observations while also taking creative leaps makes it the nexus of innovation in theory. Abductive inferences are the places where we can have real discussions on how to connect practice to theory.

In HPE we often do not seek total consensus, but rather we find places where we can “meaningfully disagree” (Bal 2002, p.13). Meaningful disagreements can happen in how we connect observations of practices to concepts that theorize these observations. After all, we only ever observe practice, we never directly observe concepts or theories, e.g., ‘professionalism’ or ‘competence’. Even calling actions ‘behavior’ is already an abductive step, in the sense that it is an interpretation of an action. Theorizing HPE hinges on operationalizations of complex concepts. For instance, reflection, professionalism, empathy, attitude, and competence are all connected to observable performance. Yet, the connection between observable performance and these abstractions is often highly problematic. For instance, we cannot *in*duce that a trainee who scores high on a reflection test actually reflected, nor can we *de*duce that trainees who say ‘I am sorry to hear that’ during a consultation are truly empathic (de la Croix and Veen 2018; Laughley et al., 2020; Veen et al. 2020).

The point here is not to decry making these kinds of connections, but to argue that we cannot treat them as obvious and given. Operationalizing concepts, i.e., translating reflection into a construct that can be observed, such as a certain type of writing, is an abductive step. I would argue that, like all other analytic steps in the research process, abductions should be made explicit as an inferential step in our research. This would not only make assumptions behind HPE theories (positionality) more explicit, but also enable other researchers and practitioners to abduce from the specific circumstances of *your* research into the unique context of their own institution.

## The art of the possible

Abductive reasoning is something we already do all the time. We might therefore wonder, why pay any attention to it if we do it anyway? This is because mostly, we do not treat abduction *as* abduction, but rather consider reasoning as either deduction or induction. In other words, acknowledging abduction has to do with the status of theory; whether we expect it to give us certainty or not, and the relationship between theory and the one who theorizes. If we conceptualize an entity who theorizes as a purely rational being, that being can only deduce or induce. However, if we conceptualize that same entity as embodied, embedded, and affective, they are abducing.

Like speaking or walking, things we do naturally but would be hard pressed to explain quite how we do them, abduction is challenging to describe and explain. Yet I can offer some concrete actions. For one thing, I think we should avoid purely deductive or inductive statements regarding theories in HPE research. For instance, instead of starting a deductive study with ‘reflection is…’ or concluding an inductive study with ‘therefore, reflection is…’, I suggest formulating such statements as hypotheses or even invitations. ‘Given our literature, we propose a definition of reflection *as* …’, or ‘on the basis of our analysis of these interviews, we suggest reflection can be seen *as*…’. While philosophy can have great value for HPE (Veen and Cianciolo 2020), in HPE research we are not in the business of making ontological claims about reality, but rather of theorizing it in a way that contributes to high-quality HPE. Peirce argued that the value of theory is not in whether it is ‘true’ in an abstract sense, but in whether it is helpful in practice.

If a theory is not the final say on the matter, but rather a step on the way to a better (but never ultimate) understanding, we should remain open to alternatives. Looking back at the history of science, including medical science, there have been many instances when what were thought to be absolute certainties turned out to be quite wrong. Aristotle believed that heavy objects fall faster than light objects, until Copernicus the consensus was on a geocentric model of the universe, and just the history of cancer research has been described as “trial and error on a giant human scale, with the emphasis at times distinctly on error”(Mukherjee 2010) - the most pertinent example perhaps being radical mastectomy, disfiguring operations based on a faulty conceptualization of cancer as a local problem.(Pawson 2018) Given HPE research is still a very young field there are likely to be many of these in our current theories of teaching and learning. Theories are, after all, viewpoints to which we are more or less committed, and that help us make informed decisions about what we should do and expect to achieve by doing it. If we depend on consensus theory, then there are no other possible explanations. An abductive approach to theory allows us to remain open to other explanations and ready and able to acknowledge our blind spots. No matter how good a theory is, we always need to be careful not to say ‘…and besides that, nothing’. Theory is never *the* theory, but *a* theory.

This leads me to a final reflection on theory and the role it might play in HPE research. In the introduction to this series, Varpio and Ellaway (2021) describe approaches on a threefold axis of the ontological, the axiological, and the epistemological. If all theory is abductive, then all theory is, in some sense, axiological. We do not randomly generate hypotheses, but we choose the ones that we prefer, at least in part, because of our values and what we deem ‘most likely’. In doing so, abduction ventures beyond merely describing what is the case, but rather, imagines what *might* be the case. Theorizing is a creative act that we need to conduct with the awareness that our creativity depends on our positionality. In the words of a theoretical physicist, “theory is the art of the possible” (Icke 2014, p.14). Once we have imagined what is possible, we can systematically test and describe the possible, i.e., have it engage in a dialogue with practice. There are theories that tell us how we should carry out our research and interpret our findings. However, there are no theories to tell us which kinds of theories we should choose in given cases. Rather, proposing *this* approach in *this* research is a creative act: an expression of what the researchers propose will lead to positive innovation in the field.

The paradoxical nature of research in HPE, which is not unique to this domain, is that conclusions from research are provisional, while the courses of action they propose for HPE practice are final. Abduction is a process of taking creative leaps from empirical observations, with the acknowledgement that there are always other possible theories and explanations. Abduction, therefore, is an open invitation to take our imagination seriously in HPE research while maintaining a sense of humility.
